# Incidence and Characteristics of Alcohol-Based Hand Sanitizer Ingestion in Florida before and during the Coronavirus Pandemic

**DOI:** 10.5811/westjem.20814

**Published:** 2025-03-24

**Authors:** Justin Arnold, Amira Athanasios, Diep Nguyen, Rahul Mhaskar

**Affiliations:** * University of South Florida, Department of Emergency Medicine, Tampa, Florida; † Hackensack Meridian Jersey Shore University Medical Center, Department of Psychiatry, Neptune, New Jersey; ‡ University of South Florida, Department of Child and Family Studies, Tampa, Florida; § University of South Florida, Department of Medical Education, Tampa, Florida

## Abstract

**Introduction:**

Hand sanitizer use and media coverage increased throughout the coronavirus-2019 pandemic. In this study our goal was to examine and compare the incidence, demographics, and clinical outcomes of exposures to alcohol-based hand sanitizers (ABHS) before and during the COVID-19 pandemic in the state of Florida.

**Methods:**

We analyzed statewide data on all ABHS exposures in adults collected by the Florida Poison Information Network from March 1, 2015–February 28, 2020 (“pre-COVID-19” cohort) and during the COVID-19 pandemic from March 1, 2020–May 5, 2023 (“COVID-19” cohort). We performed descriptive, univariable, and multivariable analyses to assess changes in sex, age, medical outcome, and intentionality of the exposure in the pre-COVID-19 vs COVID-19 study periods, and we examined the factors associated with medical outcomes.

**Results:**

We identified 876 single-substance ingestions of ABHS, 414 in the pre-COVID-19 cohort and 462 in the COVID-19 cohort. The proportions of ABHS ingestions increased significantly during the COVID-19 pandemic in all age groups except the 25–50 age group, where it decreased. Individuals 18–24 of age and those ≥51 years showed a relative increase in both intentional and unintentional ingestions during the COVID-19 period compared to the 25–50 age group. The significant risk factors associated with more severe outcomes in exposed individuals were intentional exposures and younger age.

**Conclusion:**

Unintentional ingestions of alcohol-based hand sanitizers showed a relative increase during the COVID-19 pandemic, particularly in individuals 18–25 years of age and those ≥51. Both intentional ingestions and younger age increased the likelihood of moderate or severe outcomes. Harm reduction strategies targeted toward younger individuals and those with intentional ingestions should be considered during future pandemics.

## INTRODUCTION

Hand sanitizer use and media coverage increased throughout the coronavirus-2019 pandemic. Both the US Centers for Disease Control and Prevention (CDC) and the World Health Organization strongly advocated for alcohol-based hand sanitizer (ABHS) use to help limit the spread of COVID-19.^1^ The CDC had recommended ABHS products with at least 60% ethanol or 70% isopropanol as an antiseptic to limit the spread of COVID-19 infection.^2^ The ABHS products most commonly contain ethanol, but less commonly may also contain isopropyl alcohol or quaternary ammonium compounds.^3^

There is growing evidence within the peer-reviewed literature suggesting an increase in reported ABHS ingestion throughout the COVID-19 pandemic. The US Food and Drug Administration found a 79% increase in the number of unintentional ingestions of ABHS reported to poison control centers when comparing March 2020 to March 2019.^4^ Additionally, McCulley et al found a 36% increase in ABHS exposures in children ≤5 years of age from January–April 2020.^5^ Before the current COVID-19 pandemic, Gormley et al (2012) found that the number of new cases of ABHS ingestion increased by approximately 2,000 cases each year from 2005 to 2009, according to the National Poison Data System (NPDS), suggesting an overall increasing rate of exposure in the US population.^6^ Prior to the COVID-19 pandemic, the majority of ABHS exposures were accidental and occurred mostly in pediatric populations.^4^,^5^ Peer-reviewed case studies regarding ABHS exposures, specifically within adult populations, are largely associated with alcohol and substance use disorders.^7^–^10^ The consequences of ABHS ingestion include nausea, emesis, apnea, acidosis, confusion, ataxia, sedation, coma, and death.^11^,^12^

Given that most previously reported case series have focused on unintentional and accidental exposures to ABHS,^1^,^10^,^13^ we conducted a retrospective cohort study using statewide data collected by the Florida Poison Information Center Network (FPICN) to elucidate further the trends in intentional vs unintentional ingestions of ABHS among adult populations during a global pandemic. We assessed possible associations between increased incidence of ABHS ingestions, age and sex, intentionality, and clinical outcomes.

## METHODS

As part of America’s Poison Centers, which represents the 54 accredited poison control centers in the US, the FPICN collects data from throughout the state of Florida and automatically uploads it every 9.50 minutes in real time to the NPDS, a nationwide poison surveillance tool. The FPICN data accurately represents toxic ingestions within Florida.^14^ Our COVID-19 group included all consecutive patients ≥18 of age with ingestion of ABHS reported to the FPICN during the pandemic from March 1, 2020–May 5, 2023. (May 5 was the end date of the COVID-19 pandemic per a federal Public Health Emergency declaration.) The control population (pre-COVID-19 group) included all patients ≥18 years of age with ingestion of ABHS reported in the preceding five years from March 1, 2015–February 28, 2020. We excluded exposures with multiple substances or those with incomplete data. All records were abstracted from the FPICN database by the lead author. The University of South Florid Institutional Review Board approved the study.

We conducted a descriptive analysis to describe cases of reported ABHS ingestion and assessed cases based on age, sex, intentionality, and outcome. The yearly incidence of reported ABHS ingestion was calculated as cases per one million people from 2015–2020 and 2020–2023 using the Florida population during these periods. We used the independent means *t*-test to compare the differences in data distribution of continuous attributes (eg, age) across pre-COVID vs COVID-19 cohorts. We used the chi-square test of independence to examine whether there was a statistically significant association between categorical variables. Univariable and multivariable cumulative logistic regression assessed the relationship between risk factors and the degree of medical outcome severity.

**Population Health Research Capsule**

What do we already know about this issue?

*Alcohol-based hand sanitizer (ABHS) use increased during the COVID-19 pandemic.*

What was the research question?

*How did the characteristics and outcomes of ABHS poisonings change during COVID-19?*

What was the major finding of the study?

*ABHS ingestions proportionally increased in all age groups (except those 25–50 years of age) from 83 to 142 cases per year (71%) pre-COVID-19 compared to during the pandemic (P <0.001).*

How does this improve population health?

*Understanding the characteristics, intentionality, and outcomes of ABHS ingestion during COVID-19 can lead to targeted public health messaging and interventions in future events.*

The medical outcome severity (no effect; minor; moderate; major; and death) is standardized and was defined a priori for all poisonings by the NPDS data collection tool. Minor symptoms are defined as “minimally bothersome to the patient … and usually resolve rapidly.” Moderate symptoms are defined as follows:

[M]ore pronounced, more prolonged, or more of a systemic nature … and usually some form or treatment is or would have been indicated. Symptoms were not life-threatening[,] and the patient has returned to a pre-exposure state of well-being with no residual disability or disfigurement.

Major symptoms are defined as “life-threatening or resulted in significant residual disability or disfigurement.” Since there were only two cases of medical outcome severity of death, we excluded those two cases from the multivariable analysis. We used SAS statistical software v9.4 (SAS Institute Inc, Cary, NC) to perform all data cleaning and analyses.

## RESULTS

**Figure 1:**
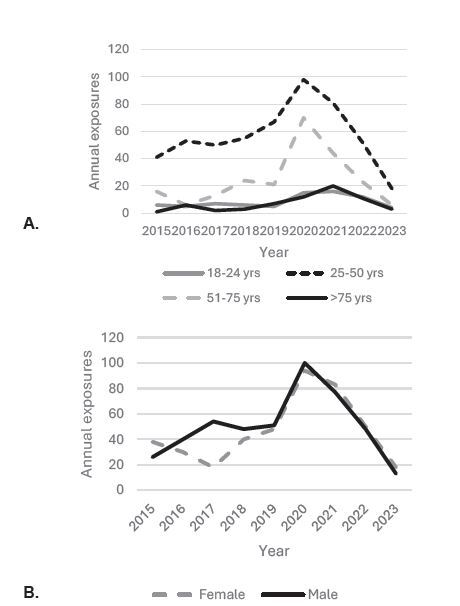
The yearly incidence of ingestion of alcohol-based hand sanitizers reported in Florida as stratified by age, sex, intention, and outcome (March 1, 2015–May 5, 2023).

We identified 876 single-substance ingestions of ABHS, with 414 in the pre-COVID-19 cohort and 462 in the COVID-19 cohort. The average annual incidence of ABHS ingestions increased considerably during COVID-19 as a whole and among all age groups (mean 83 total cases/year pre-COVID-19 increasing to a mean 142 cases/year during COVID-19). Adjusted for Florida’s population, the mean attack rate was four cases per million population in the pre-COVID-19 cohort and 6.2 cases per million population in the COVID-19 cohort. There was some variability in the number of ingestions reported to the FPICN during the COVID-19 study period with a disproportionately large increase in monthly calls during July 2020. Unintentional exposures, starting in 2019, increased significantly and exceeded intentional exposures (Table 1). The annual incidence based on age, sex, intention, and outcome are shown in Figure 1.

### Age

When assessing all individuals based on age, it appears that individuals in the COVID19 cohort were relatively older (mean age 47 years) than people in the pre-COVID cohort (mean age 42 years) (*P*-value < 0.001, Table 1). While young adults 18–24 years of age, and those 51–75 and >75 represented a larger proportion of the exposures during COVID-19, individuals 25–50 years of age showed a relative decrease in exposures to ABHS compared to other age groups (*P*<0.001, Table 1).

### Sex

There were no statistical differences in patient characteristics between the pre-COVID and COVID-19 cohorts related to sex (Table 1). Although males showed no significant changes in their use patterns, the proportion of females demonstrating no effect or minor effects increased from 74.6% during the pre-COVID-19 timeframe to 88.4% during COVID-19 (*P*<0.001, Table 3A). Additionally, females with no effect were statistically older (51.1+/−2.2 years) when compared to those with minor effects (42.4 +/−1.1 years), moderate effects (40.8+/−1.8 years), or major effects (41.4+/−4.3 years) (*P*=0.01, Table 3B). There were no deaths in any females and two deaths in males (both in the COVID-19 group).

### Intention

When assessing the effect of intentionality (unintentional vs intentional exposures vs other), the proportion of unintentional ingestions of ABHS in the COVID-19 group was higher than in the pre-COVID group, increasing from 23% of all exposures pre-COVID to 43.3% of all exposures during the COVID-19 pandemic. Still, the intentional ingestions and other ingestions groups had higher proportions during COVID-19 than before COVID-19 (*P*-value <0.001, Table 1). Intentional exposures due to “other” reasons (including adverse reactions, malicious use or tampering, withdrawal, or unknown reasons) relatively increased from 4.8% in the pre-COVID period to 8% during the COVID-19 period (Table 1). Both pre-COVID and COVID-19 cohorts demonstrated an association with unintentional ingestions and a higher mean age (51.8+/−2.4) compared to 39.9+/−0.6 years pre-COVID, 41.1+/−1.9 vs 40.8 +/−1.3 years during COVID-19, *P*<0.001).

Intentional exposures can be broadly divided into three categories: intentional suicide attempt (self-harm attempt); intentional misuse (knowingly using a product other than as directed on the label excluding psychotropic effects); and intentional abuse (using a product for recreational effects, psychotropic effects, or to achieve a high). Although intentional abuse and misuse saw a proportional decrease in exposures during the COVID-19 cohort, intentional suicide increased significantly from 29.4% in the pre-COVID cohort to 42.2% in the COVID-19 cohort (Table 2).

### Medical Outcome

During COVID-19, 34% of patients had no effect, 46.6% had minor effects, 16% had moderate effects, 3% had major effects, and there were two deaths (Table 1). There were no notable outcome differences (*P* = 0.08) in the pre-COVID vs COVID-19 groups (Table 1). However, univariable analysis showed that sex, age, being in the either the pre-COVID or COVID-19 groups, and intentional use of ABHS were all significantly related to medical outcome (Table 4). When these four variables were placed into the multivariable model with medical outcome as the dependent variable, only age and those with intentional exposures were noted to be significant predictors of medical outcomes (Table 4).

## DISCUSSION

Overall, the incidence of intentional ABHS ingestion in our study population increased statistically significantly in most age groups during COVID-19 when compared to pre-COVID-19. While we could not determine causation, the data suggests a strong correlation between the observed increase in the ingestion of ABHS and the onset of the COVID-19 pandemic. Factors likely contributing to this observed rise in ABHS ingestion include increased accessibility and visibility of ABHS; increased media attention; education on the daily recommended use of ABHS; and perhaps the absence of clear guidance regarding safe storage and use of ABHS.^4^ Online and social media trends recommending the use of ABHS in groceries and the direct ingestion of ABHS started in April 2020.

**Figure 2:**
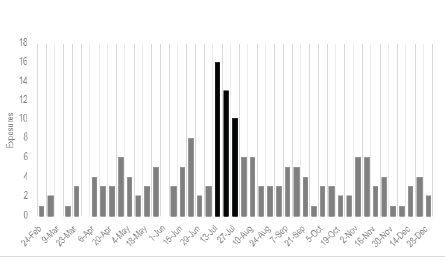
Exposures to alcohol-based hand sanitizers by week in 2020.

During the COVID-19 period two notable events in Florida may have impacted exposures to ABHS. The first was a statewide ban on ethanol sold in bars in June 2020 that lasted one week. While this may have limited ethanol access in bars, ethanol and all ABHS products remained available at grocery stores, gas stations, and liquor stores, and it likely did not have a significant impact on ABHS exposures. The second event was the methanol contamination of certain brands of ABHS in July 2020. This event was widely publicized, and exposed individuals were encouraged to contact poison centers for advice. Poison centers in Florida saw a fourfold increase in mean cases for the year during the three weeks July 13–August 2, 2020 (Figure 2), which was largely attributed to these products.

Holzman et al reported significant increases of 124% in their total reports of ABHS exposure in Arizona (intentional + unintentional) during COVID-19.^16^ Phillips et al reported a 72.5% increase in total ABHS exposures reported to Texas poison control centers during COVID-19.^17^ In Texas, the mean age of both unintentional and intentional exposures to ABHS significantly increased from 10.9 to 23.9 years of age during COVID-19. Although a similar increase in mean age was noted by Shulte et al in Texas, our subset of patients demonstrated an older baseline but a similarly significant increase in mean age during COVID-19. The mean age in the COVID-19 cohort likely increased due to a higher proportion of older adults in the 51–75 and >75-year-old categories during COVID-19 compared to the pre-COVID-19 period, as well as a baseline older population in Florida. Florida is the third most populus state in the US, with an estimated population in 2022 of 22,244,823, with 21.1% ≥65 years of age. Like Florida, Arizona has an older population (18.3% of the population ≥65 years) compared to the US overall (16.8% of the population ≥65). Our data specifically addresses patients from Florida and showed a 127% increase in the incidence of ABHS exposures from the pre-COVID-19 study period through the COVID-19 study period. It demonstrated relative increases in ABHS exposures in the younger 18–24 years of age group and individuals >51. The largest age group, 25–50, saw a proportionally decreased incidence from 67.4% in the pre-COVID cohort to 50.9% in the COVID-19 cohort.

Interestingly, although there was a decrease in minor and no effects and an increase in moderate and major effects in outcomes after exposure during the COVID-19 period in comparison with pre-COVID, while adjusting for age, sex, and intentionality, there was no statistically significant change in outcomes after exposure when comparing the pre-COVID and COVID-19 cohorts. Females were noted more likely to have either no effect or a minor effect (compared to moderate or major effects) after ABHS exposure than males during the COVID-19 pandemic (*P*<.001), but this was not the case for the pre-COVID-19 period (*P*=0.90). Although not specifically addressed in this study, this was likely due to a lower exposure dose associated with unintentional exposures. Younger adults tend to have more severe medical outcomes in univariable and multivariable analyses.

Furthermore, significant changes in the intentionality were reported to poison centers during COVID-19. As mentioned above, unintentional exposures relatively increased during COVID-19 from 23% to 43.3% of exposures, while intentional exposures relatively decreased from 72.2% to 48.3%. Traditionally, the majority (97%) of all ABHS exposures reported to US-based poison centers are unintentional exposures that occurred in children.^13^ Of those with unintentional exposures, Kweon et al reported no notable differences in the distribution of unintentional exposures, adverse reactions, or concomitant exposures in the pre-COVID vs COVID-19 study periods. Similar increased incidences of unintentional ABHS during COVID-19 were seen in children in France, Canada, and Australia.^10^

In our study, however, we focused on adults in which intentional exposures (abuse, misuse, and suicide attempts) vs unintentional exposures were more commonly reported to poison centers. When assessing the type of intentional exposure, a significant increase in the suicide attempt subset (29.4% to 42.2%) was noted, which may not be surprising due to the high level of stress and anxiety that was present during COVID-19. Intentional ingestion of ABHS was found to portend a higher risk of worse medical outcomes as compared to unintentional ingestion, likely suggesting that those with intentional ingestion (suicide attempts are often associated with larger doses) consumed a higher quantity of ABHS. However, the quantity was not analyzed in this assessment. Furthermore, those with intentional ABHS ingestions were more likely to be male and slightly younger, which may be reflective of alcohol use disorder in the general population.

Predictors of moderate or major outcomes included those with intentional ingestions and those with younger age. Intentional ingestions often involve large quantities of ABHS, which often produce more significant clinical effects, including hematemesis, central nervous system and respiratory depression, and the need for invasive monitoring or aggressive supportive care. There were two deaths reported; both were males with intentional ingestions as suicide attempts during COVID-19.

## LIMITATIONS

The limitations of this study are largely based on the limitations of poison center reporting. The FPICN records are based on voluntary, self-reported exposures by patients and physicians. Because reporting is not mandated there is potential for selection bias in reported cases. Additionally, the National Poison Database System likely under-represents our population’s total incidence of ABHS. Furthermore, given self-reported information, callers may inaccurately report the specific product or substance, dose, clinical features, and outcome. Given the variability in reporting the quantity of ABHS ingested (mostly reported as an estimated amount ingested), we did not include the quantity ingested in our analysis. Certainly, those with a higher dose are anticipated to have more severe outcomes. However, this was not specifically studied. Finally, a broad yet specific population (the State of Florida) may not apply to other populations.

## CONCLUSION

Although the ingestion of alcohol-based hand sanitizers increased broadly during COVID-19, proportional use patterns showed only slight variations compared to pre-pandemic exposures. Individuals aged 18–25 of age and ≥51 showed relatively increased rates of exposure to ABHS during COVID-19, and those with increased age demonstrated more moderate and severe outcomes. There was a significant relative increase in unintentional exposures and a relative decrease in intentional exposures, with a dramatic increase in suicide attempts among those with intentional exposures. Despite these changes, most exposed individuals demonstrated no or only minor symptoms after ingestion. The only significant risk factors associated with poor outcomes were intentional ingestion and young age. Although these findings are specific to Florida and based on Florida Poison Information Center Network data, clinicians may be able to use these findings in a similar pandemic or disaster response. Patients should be advised in future pandemics to avoid intentional ingestion of ABHS; access to resources such as counseling, suicide hotlines, poison centers, and safety campaigns should be considered. Additionally, public health messaging and education targeting younger adults may be worthwhile in mitigating these exposures.

## Figures and Tables

**Table 1:** Patient characteristics prior to coronavirus 2019 (pre-COVID group: March 1, 2015–February 28, 2020) and during the pandemic (COVID-19 group: March 1, 2020–May 5, 2023) in Florida.

Patient characteristics	Pre-COVID-19 group	COVID-19 group	P-value
Sex			0.078^a^
Male n (%)	229 (55.3)	228 (49.4)	
Female n (%)	185 (44.7)	234 (50.6)	
Age years: n (mean ± SEM)	414 (42.7 ± 0.8)	462 (47.2 ± 0.9)	<.001^b^*
Age group			<.001^a^*
18–24: n (%)	30 (7.3)	45 (9.7)	
25–50: n (%)	279 (67.4)	235 (50.9)	
51–75: n (%)	83 (20)	139 (30.1)	
>75: n (%)	22 (5.3)	43 (9.3)	
Medical outcome			0.083^a^
No effect: n (%)	120 (29)	157 (34)	
Minor effect: n (%)	187 (45.2)	215 (46.6)	
Moderate effect: n (%)	88 (21.2)	74 (16)	
Major effect: n (%)	19 (4.6)	14 (3)	
Death: n (%)	0 (0)	2 (0.4)	
Intentionality			<.001^a^*
Intentional: n (%)	299 (72.2)	223 (48.3)	
Unintentional: n (%)	95 (23)	202 (43.7)	
Other: n (%)	20 (4.8)	37 (8)	

Notes:

* indicates statistically significant with *P*-value <0.05.

a Chi-square test;

b independent means *t*-test.

*SEM*, standard error of the mean.

**Table 2:** Trends in specific intentional exposure types before COVID-19 (pre-COVID group: March 1, 2015–February 28, 2020) and during the pandemic (COVID-19 group: March 1, 2020–May 5, 2023) in Florida.

	Pre-COVID-19 group	COVID-19 group	P-value
Intentional abuse	193 (64.3%)	118 (52.9%)	
Intentional misuse	19 (6.3%)	11 (4.9%)	
Intentional suicide	88 (29.4%)	94 (42.2%)	
Total	300 (100%)	223 (100%)	0.01^a^*

Note:

* indicates statistically significant with *P*-value <0.05.

a Chi-square test of independence.

**Table:** 

**A.** Sex.			

	Pre-COVID-19 group	COVID-19 group	P-value
Female outcomes	185 (44.2%)	234 (55.8%)	0.001*
No effect	57 (30.8)	86 (36.7)	
Minor effect	81 (43.8)	121 (51.7)	
Moderate effect	39 (21.1)	18 (7.7)	
Major effect	8 (4.3)	9 (3.9)	
Death	0 (0)	0 (0)	
Male outcomes	229 (50.1%)	228 (49.9%)	0.21
No effect	63 (27.5)	71 (31.1)	
Minor effect	106 (46.3)	94 (41.2)	
Moderate effect	49 (21.4)	56 (24.6)	
Major effect	11 (4.8)	5 (2.2)	
Death	0 (0)	2 (0.9)	

Note:

* indicates statistically significant with *P*-value <0.05,

SEM, standard error of the mean.

**Table:** 

B. Sex and age.			

	N	Age: mean ± SEM	P-value
Female outcomes			< 0.001*
No effect	143	52.9 ± 1.9	
Minor effect	202	44.2 ± 1.2	
Moderate effect	57	41.1 ± 1.6	
Major effect	17	41.9 ± 3.8	
Death	0	n/a	
Male outcomes			0.02*
No effect	134	47.3 ± 1.7	
Minor effect	200	42.3 ± 1	
Moderate effect	105	42.4 ± 1.1	
Major effect	16	37.9 ± 2	
Death	2	± 18.5	

Note:

* indicates statistically significant with *P*-value <0.05,

*SEM*, standard error of the mean.

**Table 4:** Predictors of severity of medical outcomes of ABHS ingestion. of alcohol-base hand sanitizers in Florida from March 1, 2015–May 5, 2023

Predictors	Univariable analysis OR (95% CI)	Multivariable analysis OR (95% CI)	Multivariable analysis P-value
Sex (Female vs male**)	1.38 (1.08 – 1.77)	0.93 (0.72–1.21)	0.59
Age	1.02 (1.01–1.03)	1.01 (1 – 1.02)	0.027*
Group (COVID-19 vs pre-COVID**)	0.74 (0.58–0.95)	0.92 (0.71–1.2)	0.56
Ingestion reason (Intentional vs. other**)	0.55 (0.33 – 0.91)	0.56 (0.33 – 0.93)	0.03*
(Unintentional vs other**)	3.29 (1.93 – 5.59)	3.15 (1.85 – 5.36)	<0.001*

Note:

* Indicates statistically significant with *P*-value <0.05;

** indicates reference category.

*OR*, odds ratio, *CI*, confidence interval.
